# Efficacy of gelatin sponge impregnated with ropivacaine on postoperative pain after transforaminal lumbar interbody fusion: a comparative study

**DOI:** 10.1186/s12891-021-04541-w

**Published:** 2021-08-06

**Authors:** Shanxi Wang, Bo Wang, Xiaojun Yu, Tian Ma, Mubotu C. Ntambale, Jiyuan Yan, Qing Ding, Ruizhuo Zhang, Hua Wu, Chaoxu Liu

**Affiliations:** Department of Orthopedics, Tongji Hospital, Tongji Medical College, Huazhong University of Science and Technology, Jiefang Avenue 1095, Wuhan, 430030 People’s Republic of China

**Keywords:** Ropivacaine, Gelatin sponge, Postoperative pain, Transforaminal lumbar interbody fusion, Lumbar degenerative diseases

## Abstract

**Background:**

The purpose of this study was to investigate the efficacy of gelatin sponge impregnated with ropivacaine on postoperative pain after transforaminal lumbar interbody fusion (TLIF) in patients with lumbar degenerative diseases.

**Methods:**

We retrospectively reviewed patients who underwent TLIF in our department between August 2018 and January 2020. Patients were divided to ropivacaine group and saline group. A ropivacaine group whom received gelatin sponge impregnated with ropivacaine during operation, and a saline group whom were intraoperatively administered by gelatin sponge impregnated with saline. The two groups were compared in reference to postoperative hospital stay, postoperative complications and visual analog scale (VAS) scores. The consumption of postoperative diclofenac sodium suppository use was also recorded. The Oswestry Disability Index (ODI) scores and Japanese Orthopedic Association (JOA) scores were used for functional evaluation at 1 year postoperatively.

**Result:**

A total of 127 patients were evaluated in this retrospective study. The mean postoperative hospital stay in the ropivacaine group was significantly lower than saline group. The VAS score was significantly lower in patients receiving gelatin sponge impregnated with ropivacaine as compared with patients in saline group on postoperative day 1, 2, 3 and 4. The number of patients who need the administration of diclofenac sodium suppository and the mean consumption of postoperative diclofenac sodium suppository was significantly lower in the ropivacaine group as compared with saline group.

**Conclusion:**

The application of gelatin sponge impregnated with ropivacaine around the nerve root in patients undergoing TLIF can effectively control the postoperative pain and reduce postoperative hospital stay.

## Background

Transforaminal lumbar interbody fusion (TLIF) is a highly effective intervention for treating severe lumbar degenerative diseases, such as lumbar disc herniation, lumbar spondylolisthesis and lumbar spinal stenosis [1]. However, because of the spinal structure damage and nerve root traction during operation, moderate-to-severe post-surgical pain after TLIF is frequently encountered in the early postoperative period, which often leads to limitations in patient recovery and prolonged hospital stay [2, 3]. Therefore, an effective method adopted by surgeons or anesthetists is essential to relief postoperative pain and improve the comfort of patients after operation.

Ropivacaine is a long-acting amide local anesthetic with an onset time of about 10 min and a duration of 4 to 5 h. It is characterized by blocking of the sensory nerve fibers superior to motor nerve fibers, and it can cause separation block between sensory nerve fibers and motor nerve fibers even in a low concentration (0.2%) [4]. As a results, ropivacaine is increasingly used in postoperative rehabilitation of surgical patients, which can effectively block the sensory nerve for analgesia without affecting the motor function of patients [5].

In this paper, we investigated the efficacy of gelatin sponge impregnated with ropivacaine on postoperative pain after transforaminal lumbar interbody fusion in patients with lumbar degenerative diseases, so as to provide reference for clinical treatment.

## Patients and methods

### Study sample

After obtaining approval from our institutional review board, we retrospectively reviewed patients who underwent TLIF in our department between August 2018 and January 2020. The inclusion criteria were as follows: (a) lumbar degenerative diseases, including lumbar disc herniation, lumbar spondylolisthesis and lumbar spinal stenosis, (b) primary lumbar surgery, (c) with at least 1 year of follow-up. Exclusion criteria included other spinal pathology (tumor, trauma, congenital or infection) and known allergy to the ropivacaine. Based on whether they were administered by gelatin sponge impregnated with ropivacaine or gelatin sponge impregnated with saline during operation, patients were divided into ropivacaine group or saline group.

### Surgical technique

**Fig. 1:**
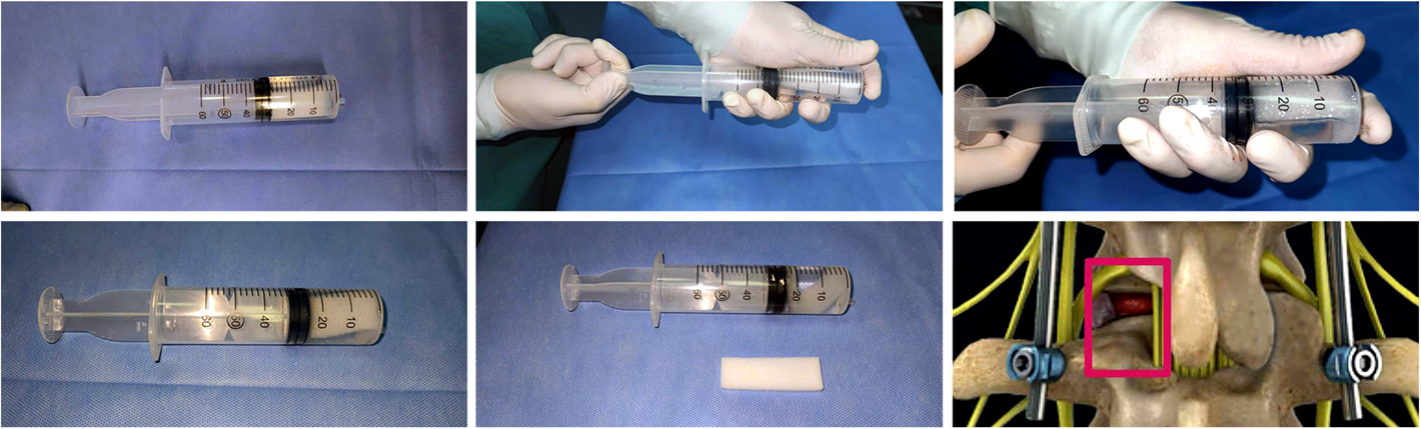
Operation steps of gelatin sponge impregnated with ropivacaine. **a** The gelatin sponge was immersed in a syringe filled with ropivacaine, and the gas in the syringe was evacuated. **b** Plug the outlet of the syringe with a finger while pulling back the piston repeatedly with the other hand to create a negative pressure in the syringe. **c** The residual gas in the gelatin sponge was sucked out by negative pressure and formed bubbles in the syringe. **d**-**e** Remove the bubbles from the syringe, the volume of ropivacaine in the syringe was significantly decreased compared to before, which proved that the gelatin sponge further absorbed ropivacaine. **f** The location of the intraoperatively gelatin sponge

All surgeries were performed under general anesthesia by one surgical team consisting of two senior orthopedic surgeons. The patient was placed prone on a radiolucent table, a posterior midline lumbar incision was performed. After exposure of bilateral lamina and facet joints, unilateral facetectomy and partial laminectomy were done to expose the intervertebral disc and achieve adequate posterior decompression. The reamer was used to remove disc tissue, and then a suitable size cage filled with the autologous bone graft was inserted into the intervertebral space. Subsequently, bilateral pedicle screws and titanium rods were installed and axially compressed to restore the lordosis, while maintaining the recovered disc height. For patients in ropivacaine group, 2 pieces of gelatin sponge (6 cm × 2 cm × 0.5 cm) impregnated with 0.75% ropivacaine were used to gently covered the surface of dura mater and nerve root. In saline group, patients received the gelatin sponge impregnated with 0.9% saline covered on the surface of dura mater and nerve root (Fig. 1). Finally, the incision was closed after placement of a drainage tube.

### Postoperative management and aftercare

The prophylactic intravenous antibiotics were administered after operation for 24 h to prevent infection. Enoxaparin (0.4 mL) was administered subcutaneously every 24 h until discharge to prevent deep venous thrombosis (DVT). The drainage was maintained for 24–48 h and then was removed. Parecoxib 40 mg every 12 h intramuscularly to control postoperative pain, and two diclofenac sodium suppositories (25 mg) were used when the patients complained pain with the visual analog scale (VAS) score [6] more than 6. No patient-controlled analgesia (PCA) pump was used in all patients. Limb functional exercises were encouraged after recovery from anesthesia, and out-of-bed activity as tolerated were encouraged after surgery.

### Outcome measurements

Patient demographic included age, gender, body mass index (BMI), diagnosis, American Society of Anesthesiologists (ASA) physical status, fusion level, fusion site, preoperative functional scores, operative time, blood loss and postoperative drainage volume were collected and compared. Clinical parameters included postoperative hospital stay and postoperative complications. Pain level was assessed using the VAS score preoperatively and on postoperative days 1, 2, 3, 4, 5 (POD 1, 2, 3, 4, 5). The rescue use of diclofenac sodium suppository was also recorded. After discharge, the patients would be followed at 1, 2, 3, 6 and 12 months and then after annually postoperatively in clinic. The Oswestry Disability Index (ODI) scores [7], and Japanese Orthopedic Association (JOA) scores [8] were used for functional evaluation at 1 year postoperatively.

### Statistical analysis

All data management and statistical analysis were performed with Statistical Package for the Social Sciences (SPSS 20.0, IBM, New York City, USA). Categorical data were tabulated with frequencies or percentages, and continuous data were expressed as the mean ± standard deviation (SD). Normality was tested using the Kolmogorov-Smirnov test. Independent t-tests were used for normally distributed continuous data and the Mann-Whitney test was used to compare abnormally distributed continuous data between two groups. Chi-square test or Fisher exact test was used to analyze the categorical variables. The level of significance was set at *p* < 0.05.

## Results

### Baseline characteristics

A total of 127 patients were evaluated in this retrospective study, which included 62 patients in ropivacaine group and 65 patients in saline group. Baseline characteristics of the patients in both groups were summarized and comparable in Table 1. There was no statistically significant differences between the two groups in terms of age, gender, diagnosis, ASA physical status, fusion level, fusion site, preoperative functional scores, operative time, blood loss and postoperative drainage volume.

**Table 1:** Baseline characteristics of the study population

	Ropivacaine (*n* = 62)	Saline (*n* = 65)	*P* value
Age (years)	53.63 ± 8.81	53.91 ± 11.60	0.879
Gender (male/female)	31/31	35/30	0.724
BMI (kg/m^2^)	23.76 ± 2.72	23.02 ± 2.68	0.128
Diagnosis			0.122
LDH	41	39	
LS	14	10	
LSS	7	16	
ASA status (I/II/III)	10/51/1	12/48/5	0.264
Fusion segments (number)	1.68 ± 0.76	1.48 ± 0.66	0.116
Fusion level (s)			0.352
1 Level	30	40	
2 Level	23	19	
3 Level	8	6	
4 Level	1	0	
Fusion site (s)			0.781
L2-L3	1	3	
L3-L4	14	13	
L4-L5	53	46	
L5-S1	36	34	
Preoperative ODI scores	62.93 ± 15.92	62.68 ± 17.70	0.931
Preoperative JOA scores	6.85 ± 3.69	7.06 ± 2.90	0.725
Preoperative VAS scores	6.76 ± 1.13	6.91 ± 1.13	0.456
Operative time (min)	208.08 ± 53.23	204.43 ± 54.23	0.703
Blood loss (ml)	314.52 ± 169.79	287.25 ± 188.34	0.394
Drainage volume (ml)	329.52 ± 229.81	383.75 ± 273.64	0.230

*LDH* Lumbar disc herniation, *LS* Lumbar spondylolisthesis, *LSS* Lumbar spinal stenosis

### Clinical outcomes

The mean postoperative hospital stay was 4.97 ± 1.43 days for the ropivacaine group and 6.23 ± 1.44 days for the saline group (*P* < 0.001). Pain scores on POD 1, 2, 3 and 4 were significantly lower for ropivacaine group compared to saline group (2.40 ± 0.86 VS. 3.18 ± 1.01, *P* < 0.001; 2.79 ± 0.94 VS. 3.85 ± 1.11, *P* < 0.001; 2.48 ± 0.90 VS. 2.95 ± 0.82, *P* = 0.003; 2.24 ± 0.82 VS. 2.58 ± 0.79, *P* = 0.018, respectively), no statistical difference was identified between two groups on POD 5. In ropivacaine group, 19 patients required the administration of diclofenac sodium suppository, and the mean consumption of diclofenac sodium suppository was 15.32 ± 28.76 mg. In saline group, the number of patients who need analgesic rescue was 34, and the mean consumption of diclofenac sodium suppository was 28.46 ± 34.20 mg. Fewer patients required diclofenac sodium suppository in ropivacaine group than saline group and the difference was statistically significant. Lower diclofenac sodium suppository consumption was found in ropivacaine group than saline group, and the difference also has statistically significant. Delayed wound healing was found in one patient in ropivacaine group and three patients in saline group, no postoperative nausea and vomiting (PONV) or DVT was found in neither group. There was no statistically significant difference between two groups in the incidence of postoperative complications. (Table 2).

**Table 2:** Comparison of postoperative hospital stay, VAS scores, requirement of diclofenac sodium suppositories and postoperative complications between the two groups

	Ropivacaine (n = 62)	Saline (n = 65)	*P* value
Postoperative hospital stay (days)	4.97 ± 1.43	6.23 ± 1.44	< 0.001
VAS scores			
POD1	2.40 ± 0.86	3.18 ± 1.01	< 0.001
POD2	2.79 ± 0.94	3.85 ± 1.11	< 0.001
POD3	2.48 ± 0.90	2.95 ± 0.82	0.003
POD4	2.24 ± 0.82	2.58 ± 0.79	0.018
POD5	2.10 ± 0.82	2.23 ± 0.64	0.254
Diclofenac sodium suppository			
Number (n)	19	34	0.019
Dose (mg)	15.32 ± 28.76	28.46 ± 34.20	0.010
Complications			
Delayed wound healing	1	3	0.619
PONV	0	0	–
DVT	0	0	–

*VAS* Visual analog scale, *POD* Postoperative day, *PONV* Postoperative nausea and vomiting, *DVT* Deep venous thrombosis

At one year after surgery, the mean ODI scores of ropivacaine group was 10.81 ± 5.52, while the mean ODI scores of saline group was 11.97 ± 6.26. The average JOA scores of ropivacaine group and saline group were 27.06 ± 1.33 and 26.71 ± 1.73, respectively. There were no statistically significant differences between two groups in the ODI scores and JOA scores at one year postoperatively. (Table 3).

**Table 3:** Comparison of ODI scores and JOA scores at 1 year postoperatively between two groups

	Ropivacaine (n = 62)	Saline (n = 65)	*P* value
ODI scores	10.81 ± 5.52	11.97 ± 6.26	0.270
JOA scores	27.06 ± 1.33	26.71 ± 1.73	0.139

*ODI* Oswestry Disability Index, *JOA* Japanese Orthopaedic Association

## Discussion

The lower back pain is a leading reason of disability worldwide, which often caused by lumbar degenerative diseases, such as lumbar disc herniation, lumbar spondylolisthesis and lumbar spinal stenosis [9]. For patients who have failed non-surgical treatment, lumbar fusion is an effective option, which not only relieves pain, but also improves the life quality of patients [10, 11]. According to the surgical approach, lumbar fusion can be divided into different types, the most common are anterior lumbar interbody fusion (ALIF), lateral lumbar interbody fusion (LLIF), posterior lumbar interbody fusion (PLIF) and TLIF [12].

TLIF was first described by Harms and developed as a modification of PLIF [13]. Compared to other approaches, it can directly access to the intervertebral foramen area, with little damage to the structural integrity of spinal [14–16]. There are also evidences show that TLIF can reduce the risk of dural tears and nerve root injury although has a lower rate of postoperative complications and better functional recovery [10, 15]. As a result, TLIF has become a well-established and prevalent surgical approach for degenerative lumbar diseases [14, 15]. However, there are also disadvantages of TLIF, one of the most major challenges is the severe postoperative pain related to the extensive muscle dissection and nerve root stimulation during operation [10]. As uncontrolled postoperative pain is directly associated to longer hospital stays, increased costs, delayed recovery and more complications, the management of postoperative pain is highly imperative for patients undergoing TLIF [17].

Although opioids or non-steroidal anti-inflammatory drugs play an important role in the control of postoperative pain for spinal patients, the side effects such as PONV, pruritus, respiratory depression or peptic ulcer still limited their use [18, 19]. Besides, excessive use of opioids can contribute to long-term opioids dependence and abuse [20]. Therefore, the control of postoperative pain is essential to curb the overuse of analgesics and related adverse outcomes. Prasartritha et al. [21] reported that epidural infusion analgesia is safe and effective for controlling postoperative pain in spinal surgery. However, the technique is sometimes very dangerous because of the possible penetration of duramater, which can injure spinal cord and cause total spinal subarachnoid anesthesia [22]. Local infiltration analgesia may be an another alternative method to control postoperative pain in spinal surgery. Tomov et al. [23] investigated the efficacy of subcutaneous infiltration of liposomal bupivacaine on postoperative pain management and narcotic use following TLIF. Although the result showed that the application of local infiltration can significantly reduce postoperative pain and the consumption of postoperative analgesic, it however does not significantly reduce the length of hospital stay.

Patients undergoing spinal surgery may experience intense pain in the early postoperative period, the use of gelatin sponge can slowly release ropivacaine around the nerve root so as to extend the postoperative analgesia time [2, 24]. In most studies, ropivacaine was injected into gelatin sponge by syringe [8, 25]. However, because of the residual gas in the gelatin sponge, it is difficult to make the gelatin sponge completely absorb ropivacaine in this method. We created a negative pressure in the syringe, which can effectively exhaust the residual gas in the gelatin sponge, so that the gelatin sponge can absorb enough ropivacaine, thereby increasing the release time of ropivacaine and prolonging the postoperative analgesia effect. Our results showed that the application of gelatin sponge impregnated with ropivacaine could significantly reduce the postoperative pain on POD 1, 2, 3 and 4, especially on POD 1 and 2, the VAS scores of ropivacaine group was markedly lower than saline group. Fewer patients in ropivacaine group need the administration of diclofenac sodium suppository, and the mean consumption of diclofenac sodium suppository was also less in ropivacaine group. Besides, our research also suggested that the application of gelatin sponge impregnated with ropivacaine during operation can effectively shorten the postoperative hospital stay, which may be due to the limitation of postoperative pain. The control of postoperative pain is conducive to the recovery of postoperative function in spinal patients, and the early out-of-bed activity is beneficial to the prevention of pulmonary complications and deep venous thrombosis. In addition, a shorter length of postoperative hospital stay means that the costs of hospitalization can be saved.

There are several limitations to our study. One of the limitations is that this was a retrospective study. A randomized controlled study is needed to further investigated the efficacy of gelatin sponge impregnated with ropivacaine on postoperative pain after transforaminal lumbar interbody fusion in patients with lumbar degenerative diseases. Secondly, since all surgeries in this study were performed by one surgical team including two senior orthopedic surgeons at a single center, multi-center research is needed to further verify our conclusions. Furthermore, further study is required to compare the efficacy for gelatin sponge impregnated with ropivacaine and other analgesic strategies on postoperative pain in patients treated with TLIF.

## Conclusions

Our study demonstrated that the application of gelatin sponge impregnated with ropivacaine in patients undergoing TLIF can effectively control the immediate acute postoperative pain and reduce postoperative hospital stay.

## Data Availability

The data sets generated and/or analysed during the current study are not publicly available due to limitations of ethical approval involving the patient data and anonymity but are available from the corresponding author on reasonable request.
